# Evidence for possible association of vitamin D status with cytokine storm and unregulated inflammation in COVID-19 patients

**DOI:** 10.1007/s40520-020-01677-y

**Published:** 2020-09-02

**Authors:** Ali Daneshkhah, Vasundhara Agrawal, Adam Eshein, Hariharan Subramanian, Hemant Kumar Roy, Vadim Backman

**Affiliations:** 1 Department of Biomedical Engineering, Northwestern University, Evanston, IL USA; 2 Boston Medical Center, Boston, MA USA

**Keywords:** COVID-19, Vitamin D, Cytokine storm, C-reactive protein, Case mortality ratio, SARS-CoV-2

## Abstract

**Objectives:**

We present evidence for a possible role of Vitamin D (VitD) deficiency in unregulated cytokine production and inflammation leading to complications in COVID-19 patients.

**Design:**

The time-adjusted case mortality ratio (T-CMR) was estimated as the ratio of deceased patients on day *N* to the confirmed cases on day N-8. The adaptive average of T-CMR (A-CMR) was calculated as a metric of COVID-19 associated mortality. A model based on positivity change (PC) and an estimated prevalence of COVID-19 was used to determine countries with similar screening strategies. A possible association of A-CMR with the mean concentration of 25-hydroxyvitamin D (25(OH)D) in elderly individuals in countries with similar screening strategy was investigated. We considered high C-reactive protein (CRP) in severe COVID-19 patients (CRP ≥ 1 mg/dL) as a surrogate of a cytokine storm. We considered high-sensitivity CRP (hs-CRP) in healthy subjects as hs-CRP ≥ 0.2 mg/dL.

**Results:**

A link between 25(OH)D and A-CMR in countries with similar screening strategy is evidence for VitD’s possible role in reducing unregulated cytokine production and inflammation among patients with severe COVID-19. We observed an odds ratio (OR) of 1.8 with 95% confidence interval (95% CI) (1.2 to 2.6) and an OR of 1.9 with 95% CI (1.4 to 2.7) for hs-CRP in VitD deficient elderly from low-income families and high-income families, respectively. COVID-19 patient-level data show an OR of 3.4 with 95% CI (2.15 to 5.4) for high CRP in severe COVID-19 patients.

**Conclusion:**

We conclude that future studies on VitD’s role in reducing cytokine storm and COVID-19 mortality are warranted.

## Introduction

The recent global outbreak of COVID-19 imposed catastrophic impacts on every society, specifically among elderly populations. Currently, no treatment or vaccine against the virus is available. Consequently there is a significant need to elucidate potential approaches that can reduce the number of severe COVID-19 cases and associated mortality.

Large-scale data show that the mortality rate of COVID-19 varies dramatically across countries. For example, a higher case fatality ratio has been reported in Spain, Italy, and the UK compared to that in the US and Germany. The cause for these disparities is not well understood. Several hypotheses have been proposed, including the emergence and circulation of different strains of the virus [1–3], idiosyncrasies in COVID-19 testing strategies, quality and access to health care, demographic variables such as the prevalence of elderly within a given population, and socioeconomic factors [4]. Some studies have suggested an analysis of age-specific case fatality ratio (CFR) and time-adjusted case mortality ratio (T-CMR) for a more insightful study of COVID-19 infection [5, 6]. Initial reports and data obtained from various studies suggest that the elderly population are disproportionately impacted by COVID-19 [7]. The substantially higher CFR of the elderly population thus compels an age-specific analysis of COVID-19 data.

Aging can lead to a weakening of the innate immune system [8] which may play a role in the development of severe COVID-19. Specifically, a weak innate immune system response in the elderly can lead to a higher load of SARS-CoV-2 and a consequent overactivation of the adaptive immune system, leading to an increased level of cytokine production [9]. Clinical data obtained from COVID-19 patients in China showed high concentrations of cytokines and possible cytokine storm in patients admitted to the ICU [10].

The role of VitD in regulating the immune system has been supported by multiple studies [11]. VitD can suppress cytokine production by simultaneously boosting the innate immune system (thus reducing the viral load) and decreasing the overactivation of the adaptive immune system to immediately respond to the viral load. Some researchers have suggested the potential role of VitD in suppressing cytokine storm during the 1918–1919 viral influenza pandemic [12]. Moreover, the role of VitD in enhancing immune response in flu and previous coronaviruses has been suggested [11, 13]. Studies have further shown VitD’s importance for protection against different infections [14] including respiratory tract infections [15–17]. VitD delivers such a protection through the regulation of the immune system via VitD receptors [18] and in this process it also reduces the production of pro-inflammatory cytokines [19–21]. Recent data have shown a strong correlation between excessive cytokine production and severity of COVID-19 [10]. It is this ability of VitD in suppressing cytokine production [22, 23] that motivated our focus on VitD deficiency and its association with severe COVID-19.

To the best of our knowledge, no randomized blinded experiment has yet reported VitD status and cytokine levels in patients with COVID-19. In spite of this, we investigated a possible association between VitD status and unregulated inflammation [24, 25] such as C-reactive protein (CRP) which is a surrogate of cytokine storm [26].

CRP is produced primarily in the liver in response to inflammation to minimize damage to tissues from autoimmunity, infection, and other causes. It is a nonspecific marker and is partially elevated via the bioactivity of cytokines such as interleukin (IL)-6 [27]. Pro-inflammatory cytokines become a major contributor to production of CRP during cytokine storm in COVID-19 infection. 25-hydroxyl vitamin D3-1α-hydroxylase (CYP27B1) plays an important role in metabolizing VitD into calcitriol, the active form of VitD. Calcitriol binds and activates VitD receptor (VDR) in the nucleus, and controls gene expression [28]. Expression of VDR and CYP27B1 can reduce the inflammatory markers [28, 29]. Together, this suggests a possible impact of VitD on decreasing pro-inflamatory cytokine production and CRP.

Here we combine VitD and high-sensitivity CRP (hs-CRP) data from NHANES, 2009–2010 dataset with clinical data from COVID-19 patients [31] to assess a possible role of VitD in regulating inflammation and cytokine production which is a major risk factor for severe COVID-19 across different countries.

## Methods

COVID-19 affected, and deceased cases were obtained from Kaggle [32] as of April 21, 2020 except the data from England which was provided by the UK government [33]. Worldometers.info was used as an independent source to crossvalidate the data. Testing data were obtained from Our World in Data and publicly available official national reports for each country [34, 36]. Different national reports and published articles were used to estimate the age distribution of the hospitalized COVID-19 patients in the US [37, 38], France [39], Italy [40], Switzerland [41, 42] the UK [43], and Spain [44]. The ratio of deceased to hospitalized patients was obtained and estimated from national reports and published articles for Italy [40], Spain [44], Iran [45], the UK [43], and France [39]. Data associated with the number of hospital beds in each country were obtained from WHO [46] and those on the number of critical care beds were obtained from recently published articles [47, 48].

The concentration of 25-hydroxyvitamin D (25(OH)D) among the elderly population in each country was obtained from prior studies [49–54, 61–65]. CRP, VitD, and demographic variables data of the subjects were pooled from the cross-sectional data from 2009–2010 NHANES, conducted by the National Center for Health Statistics (NCHS), Centers for Disease Control and Prevention (CDC) [55]. Data on blood pressure [56], body to mass ratio [57], and diabetes [58], were obtained from published articles. Data on coronary heart disease (CHD) death rates were obtained from World Life Expectancy [59]. The link between high CRP and severe COVID-19 was examined based on data from a study assessing the characteristics of COVID-19 patients in China [31]. The T-CMR is defined as the estimated ratio of deceased patients on day *N* (*D*_*N*_) to confirmed patients on day N-8 (*C*_N-8_). Adaptive averaging of T-CMR (A-CMR) was calculated based on a weighted average technique as shown in Eq. (1).

1 $${\text{A}-\text{CMR}} = \mathop \sum \limits_{n = 1}^{n = N} a_{n} \times {\text{ T}-\text{CMR }}\left[ n \right],a_{n} = c_{n} /\mathop \sum \limits_{i = 1}^{i = N} c_{i} ,$$

where *N* is the number of days with more than 10,000 confirmed cases in the country (except in S. Korea where the threshold is 5,000), *c*_*i*_ is the number of confirmed cases at day *i*, T-CMR (*n*) is T-CMR on day *n*, and *a*_*n*_ is a coefficient that describes the weight of T-CMR on day *n*. Positivity change (PC) is calculated using a moving average of size 5 on the ratio of new confirmed cases to the new tested individuals on day *N* as shown by Eq. (2).

2 $${\text{PC}} = \mathop \sum \limits_{i = 1}^{i = 5} 0.2 \times \left( {C_{N + 1 - i} {-} \, C_{N - i} } \right)/(T_{N + 1 - i - } T_{N - i} ),$$

where *C*_*N*_ is the total confirmed cases on day *N* and *T*_*N*_ is the total number of tested cases on day *N*. The starting point of each curve is the day that the country reported at least 10,000 patients in total (except S. Korea > 5,000). Elevated levels for hs-CRP was defined as hs-CRP ≥ 0.2 mg/dL among healthy subjects (threshold suggesting low-grade inflammation and risk of cardiovascular disease [25]) and high CRP for COVID-19 patients was defined as CRP ≥ 1 mg/dL (indicating high-grade inflammation). Low-income and high-income families were determined based on a variable calculated by dividing the total income of a family by a poverty index which was calculated based on guidelines described by the Department of Health and Human Services’ (HHS)), considering factors such as family size, state, and year [55]. In our analysis, VitD and CRP data for 4526 subjects with an income to poverty index between 0 and 2 are associated with low-income families while 3819 subjects with an higher Index greater than or equal to 2 are associated with high-income families.

## Results and interpretation

### COVID-19 fatality

**Fig. 1:**
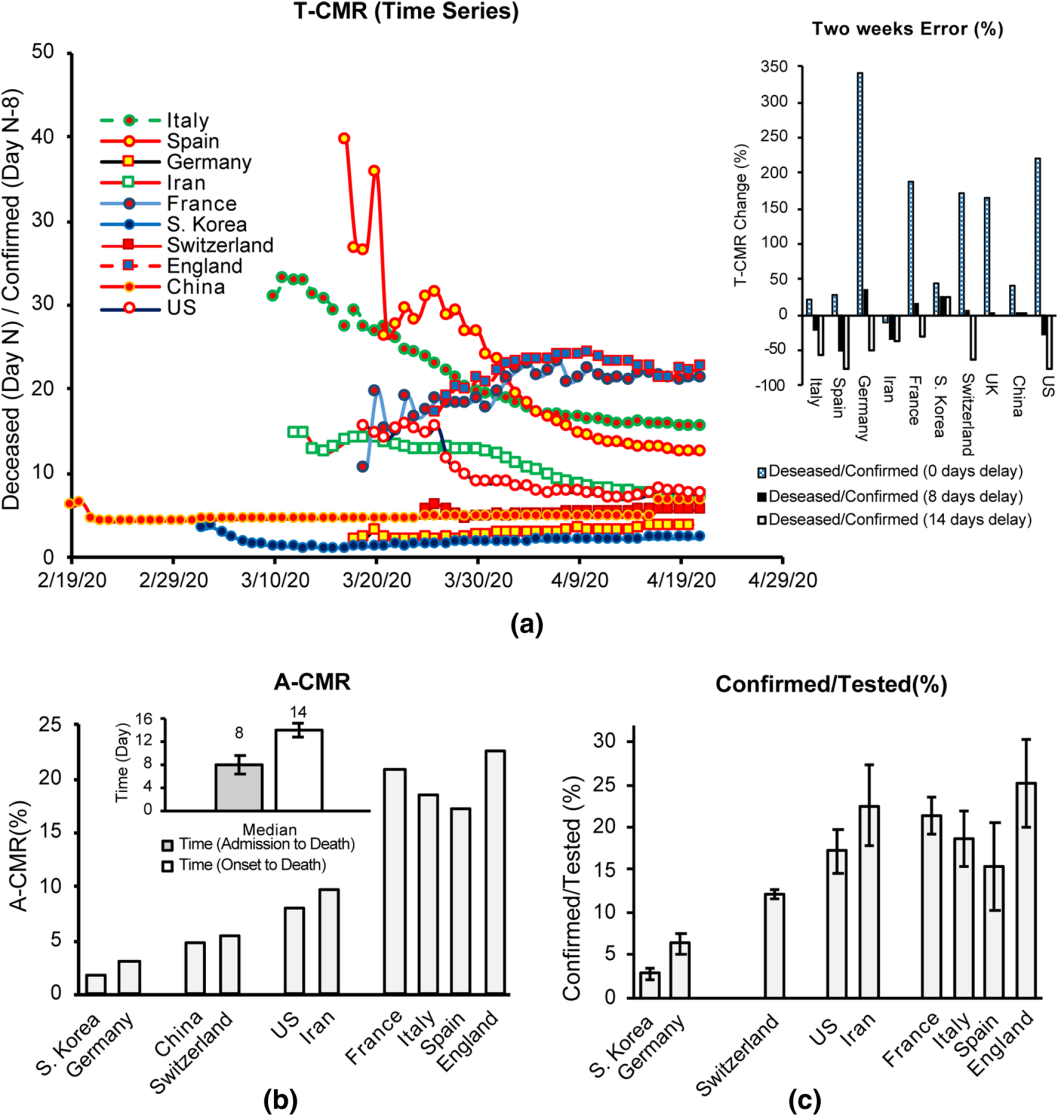
**a** T-CMR (8 days) as of April 21. A 2-week variability (100 × (T-CMR_April 20_ – T-CMR_April 6_)/T-CMR_April 6_) calculated at different T-CMR delays of 0 days, 8 days and 14 days. **b** A-CMR as of April 21 [26]. **c** Percentage of confirmed to tested ratio suggests an impact of screening strategy on A-CMR [32, 36]. France testing data are the number of tests [34]. England reported the number of tests (from April 1, 2020–April 21, 2020) and we estimated number tests before April 1, 2020 by multiplying the number of patients by 1.24 (the ratio obtained from average ratio of number of tests to the number of patients from April 1, 2020, to April 7, 2020 [33]. US data are mainly the number of people tested (some labs have reported the number of tests) [34]. Iran and Spain testing data are estimated from two reported statements by public authorities [34–36]

Ambiguity as to the incubation period of COVID-19 makes the calculation of the true mortality rate for the disease a challenging task [5, 6]. Bureaucratic screening policies, as well as demographic and cultural variables further increase the difficulty of estimating disease onset and calculating an accurate case mortality rate (CMR). Analysis of time events reported from 41 deceased patients in Wuhan (Hubei, China) shows a median time of 8 days between admission and time of death, and 14 days between the onset of symptoms and time of death (shown in the inset in Fig. 1a) [60]. This suggests a delay between the time the confirmed cases are reported and the time deceased patients are counted. In other words, the total number of deceased patients at day *N* (*D*_*N*_) is attributed to the total number of confirmed patients at day N-8 (*C*_N-8_) which is equal to the total number of cases at the onset of the symptoms on day N-14 (*O*_N-14_). Time adjusted-CMR (T-CMR) with a delay of 8 days (*D*_N_/*C*_N-8_) is therefore used in this study (shown in Fig. 1a). Calculating the percent difference between T-CMR on April 20 and April 6 for three different delays of 0 days, 8 days and 14 days suggests that an 8-day delay presents the least variation across countries. Figure 1a shows time series data for T-CMR drifting for some countries. Intense variations in the ratio of confirmed to tested patients can change the results for T-CMR over the course of the pandemic for multiple reasons. With the deaths of the most vulnerable members of a population, T-CMR is expected to decrease over time. In addition, increasing screening capabilities will increase the chance of identifying mild cases, thus reducing T-CMR. As a result, different values for T-CMR are calculated throughout the pandemic and the question arises of which value is more representative of the intrinsic mortality characteristic of the virus within each country.

#### A-CMR

T-CMR varies each day and this increases its uncertainty to represent overall mortality of the virus. To calculate a more accurate estimate of overall mortality of the virus, we created a framework based on two factors. First, we considered only outbreaks of 10,000 confirmed patients or greater (except in S. Korea where the threshold was set to 5,000 as the total confirmed cases stayed below 10,000 until April 3, 2020) to provide a reliable T-CMR. Next, an average of the T-CMRs was calculated given a higher weight for the T-CMRs that represent a higher population. A-CMR for each country is calculated using Eq. (1) and the results (shown in Fig. 1b) suggest varying A-CMR values across countries.

S. Korea and Germany report a comparably low A-CMR of 1.8% and 3.1%, respectively. The A-CMR in Switzerland (A-CMR = 5.3%) and China (A-CMR = 4.9%) is higher than in S. Korea and Germany but is lower than in the US (A-CMR = 8%) and Iran (A-CMR of 9.7%). Spain (A-CMR = 17.3%), Italy (A-CMR = 18.5%), France (A-CMR = 20.9%) and England A-CMR = 22.5%) report the highest A-CMR. Multiple factors may contribute to the difference in A-CMR across these countries. Figure 1c shows the average ratio of confirmed (*C*) to tested (*T*) cases in each country. Comparison of Fig. 1b, c shows that countries with mass screening strategies (low *C*/*T* ratio) report a substantially lower A-CMR than other countries. One reason could be that countries with an aggressive screening policy tend to detect more cases of mild, less deadly COVID-19 and will thus report a lower A-CMR, as mild COVID-19 cases are generally not fatal. We consider positivity (C/T) or PC to be a better indicator of the impact of screening strategy than total tests per capita. The reason being that higher testing is required when the prevalence of COVID-19 increases.

#### Screening status

**Fig. 2:**
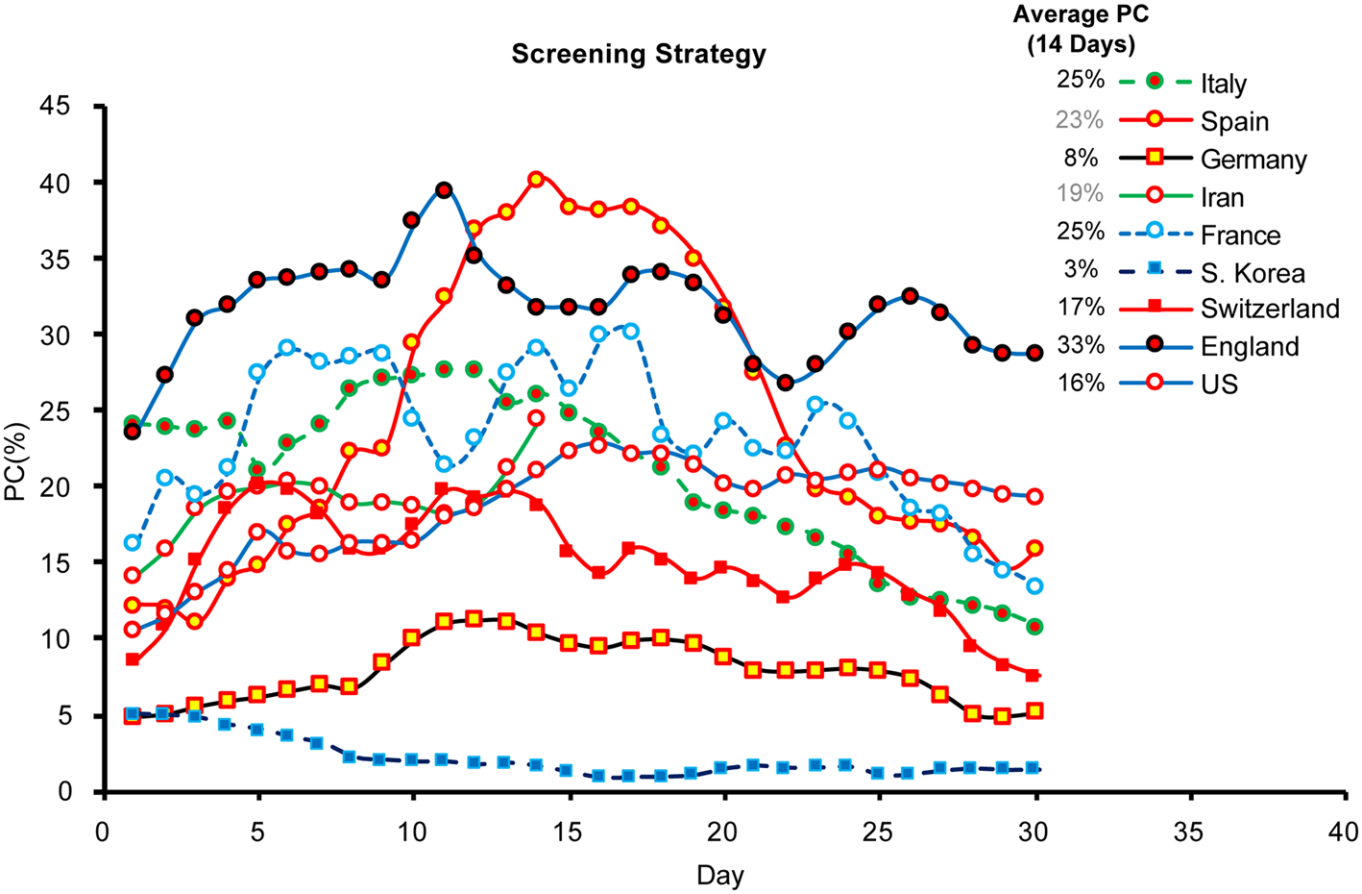
PC over time compares growth rate of COVID-19

It is important to control for screening strategies and age distribution across countries before comparing VitD status, as such variables may impact A-CMR. Two factors can be used to evaluate the screening strategies in different countries; (1) PC, and (2) the prevalence of COVID-19. We first calculated the PC to illustrate the variation in positivity in different countries over time in Fig. 2. The average PC value in the first 14 days is calculated and the results are shown in the inset of Fig. 2. Based on this analysis, we observed that S. Korea, Germany, and Switzerland have lower PC values, while Iran, the US, France, Italy, Spain, and England share higher PC values.

**Fig. 3:**
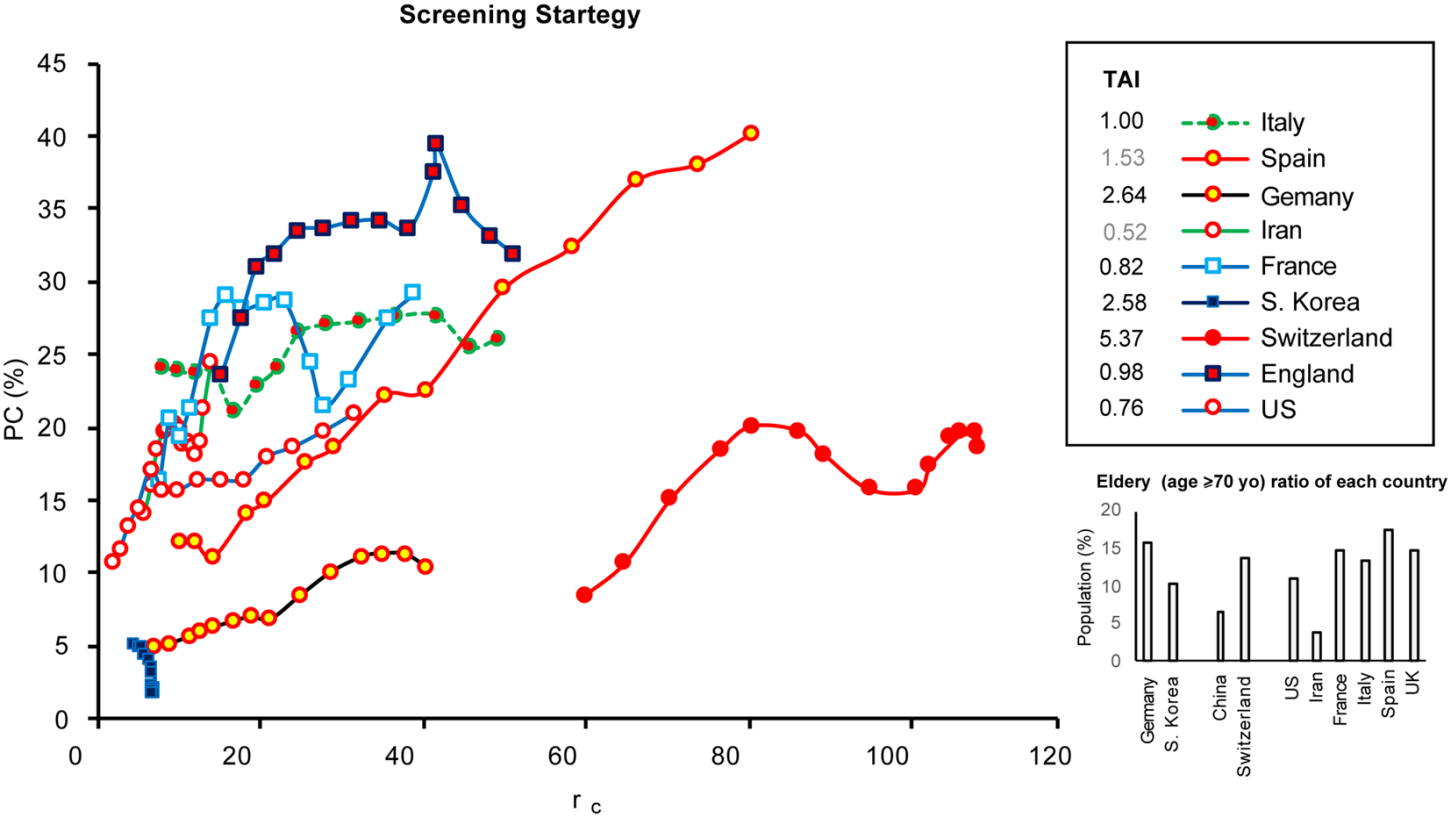
PC against *r*_*c*_ for 2 weeks after each country reaches 10,000 patients (except S. Korea > 5,000 patients)

As positivity depends on the prevalence of COVID-19, we extended our analysis by evaluating PC as a function of prevalence. We calculated an average number of confirmed cases per one million population per day over 21 days (*r*_*c*_) and used it as an indicator of the prevalence of COVID-19 in each country. We plotted PC against *r*_*c*_ for 2 weeks in Fig. 3 and the results suggest that the countries are clustered into two major groups where a more aggressive screening strategy is used such as in S. Korea, Germany and Switzerland compared to Spain, Italy, France, the England, the US, and Iran. A testing aggressiveness index (TAI) is calculated using Eq. (3) which presents a quantitative illustration for Fig. 3.

3 $${\text{TAI}} = \mathop \sum \limits_{n = 1}^{n = 14} r_{c} \left[ n \right]/{\text{PC }}\left[ n \right], \, r_{c} \left[ n \right] \, = \, (C_{n} {-} \, C_{n - 21} )/P,$$

where *P* is the population in millions of the countries, and *C*_*n*_ is the total number of confirmed patients on day *n*. TAI values for each country are presented in the inset in Fig. 3.

A small TAI is associated with a large delta in PC and a small delta in prevalence which indicates the population of the tested subjects (associated with PC) does not represent the number of confirmed subjects across the country’s population, thereby indicating a less aggressive screening strategy. The quantitative illustration of TAI suggests a more aggressive screening status (1.00 < TAI < 5.37) in Germany, S. Korea, and Switzerland, and a less aggressive screening status in Spain, Italy, France, England, the US, and Iran (0.51 < TAI < 1.54). The least aggressive screening status is found in Iran with TAI of 0.52. It should be noted that Spain and Iran have reported complete and confirmed patient information but limited data on testing cases. The screening data from Iran and Spain are estimated from only two testing data points with an average test rate reported by the public authorities. The limited number of data points may increase the error in our estimation, which is why these presented results are highlighted in gray. Furthermore, age distributions of different countries are shown in the inset of Fig. 3 and suggest a similar age distribution between the US, England, France, Spain, and Germany.

#### Possible effect of VitD on A-CMR

Screening status and the age distribution notably impact A-CMR among the population. We evaluated the possible association of A-CMR with VitD in countries with similar screening strategies.

#### Countries with less aggressive screening status

**Fig. 4:**
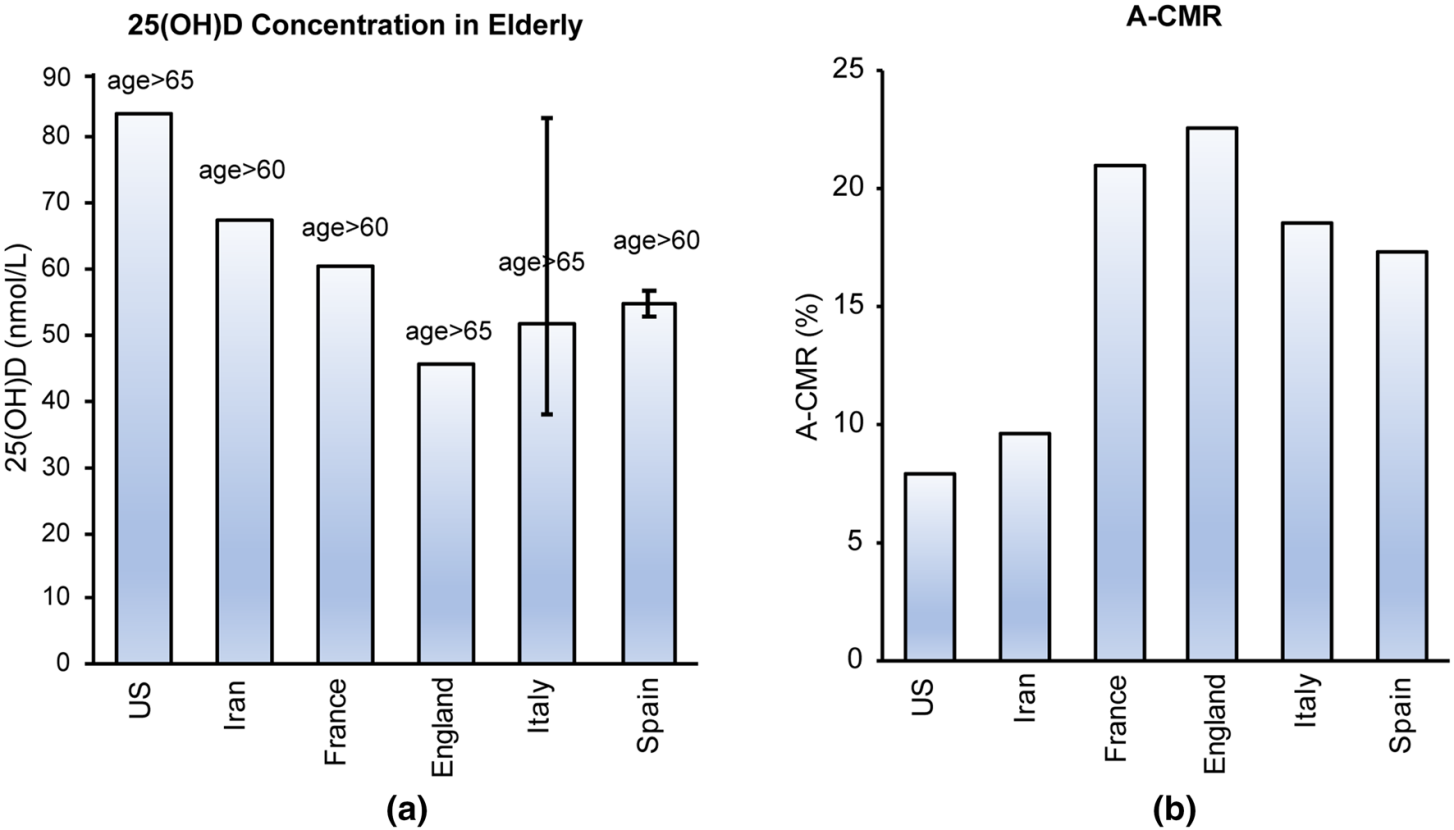
**a** Mean 25(OH)D in the elderly population in the US [61], Iran [62], France [63] and the UK [64]. Estimated 25(OH)D concentration of elderly in Italy [49–51, 65], and Spain [52]. **b** A-CMR for the US, Iran, France, England, Italy, and Spain

The 25(OH)D concentration among the elderly (age > 60 yo or age > 65 yo) in countries with less aggressive screening policies are shown in Fig. 4a. A comparison of the A-CMR and the mean 25(OH)D concentration suggests an inverse relationship between A-CMR and 25(OH)D concentration. In particular, the elderly population in the UK presents the lowest mean 25(OH)D level while England, which consists of over 90% of total COVID-19 deceased cases in the UK, reports the highest A-CMR. The US with the highest mean 25(OH)D in elderly also reports the lowest A-CMR. Iran and France, countries with higher mean 25(OH)D concentration than the UK, report a lower A-CMR. The age distribution of the elderly among these countries, shown in the inset in Fig. 3, indicates the US, France, and the UK have a similar elderly ratio while Iran and China have a lower elderly population than others.

The concentration of 25(OH)D in the elderly in Iran is estimated based on the data from 128 elderly in the city of Isfahan (geographically located in the middle of the country) as 67.5 nmol/L over the year. This result is in agreement with the comprehensive, epidemiological and ecological descriptive study on Vitamin D Status of adults in Iran (308,005 People, from 2009–2018) which estimated a mean 25(OH)D concentration of 63.5 nmol/L during the most recent year of 2018 [66]. 25(OH)D concentration in France is estimated as 60.5 nmol/L in 2011–2012 which is in agreement with the estimation of 57.5 nmol/L during 2005–2006 from a previous study [67]. An estimate of 25(OH)D concentration from Italy and Spain is also included in this figure. The two studies investigating the 25(OH)D concentration among elderly in Spain has reported a median concentration of 25(OH)D instead of mean. This is the case for one out of the four studies reporting 25(OH)D in Italy as well. Due to this estimation, error bars have been used to describe the range of the concentration of 25(OH)D reported for these two countries. Studies involving different cohorts (the Asturias study and the Pizarra study) in Spain estimated a slightly different concentration of 25(OH)D among the Spanish population. This led us to estimate a median concentration between 53 nmol/L to 59.5 nmol/L (values estimated from a figure from the published study) [52]. The variation of reported 25(OH)D concentration of the elderly population in Italy was concerning. A study of 13,110 adults in Northwestern Italy estimated the median 25(OH)D concentration of 47 nmol/L among the elderly living there [49], while another study using data from 2,694 community-dwelling elderly from Northern Italy (results from the Progetto Veneto Anziani study) estimated a mean 25(OH)D concentration of 83 nmol/L [50]. A third study of 449 elderly women (age > 65 yo) in southern Italy estimated a mean 25(OH)D concentration of 37.7 nmol/L [51] and another population-based cohort study of 1006 elderly (age > 65 yo) in northern Italy (Tuscany region) estimated median 25(OH)D concentration of 40 nmol/L [65].

#### Countries with aggressive screening status

Our analysis and calculation of TAI indicates Germany, Switzerland and S. Korea have a more aggressive screening strategy. Thus, the link between 25(OH)D and A-CMR for these three countries were investigated. A rigorous population-based study of VitD in 1418 elderly during 2009–2010 in Germany suggests 25(OH)D concentration of 51.5 nmol/L for the elderly population during the year where the lowest concentration of 39 nmol/L was observed in March and the highest concentration of 64 nmol/L was observed in August [68]. Analysis of 25(OH)D concentration of 1816 elderly age > 65 yo in Germany from 2008 to 2011 by a separate research group suggests a mean concentration of 43.2 nmol/L (41 nmol/L in females and 45 nmol/L in males) [53]. Both these studies have shown a notable variation of mean 25(OH)D (over 60%) between March and August in Germany. The difference in distribution samples collected in different seasons can impact the estimated concentration of 25(OH)D. In our analysis, we estimated the 25(OH)D in the elderly population in Germany as the weighted average of the two data as 47.3 nmol/L, and the error bar describes the difference between these two values. VitD data in S. Korea are reported from a study that analyzed data of 7196 elderly age > 65 yo from 2008 to 2014 [54]. The data from this study are collected uniformly across different seasons in each year [54]. A SENECA study of 153 elderly, 77 yo to 82 yo, in Switzerland estimates a mean 25(OH)D concentration of 43.2 nmol/L within December and March [69]. A small number of samples and the fact that the samples have not been collected throughout the year does not allow for an accurate comparison of this data with the data obtained from Germany and Switzerland. A recent rigorous analysis of 25(OH)D in 1818 subjects with a mean age of 56 yo (14–94 yo) in Switzerland suggests a lower concentration of 41.6 nmol/L in March and a higher concentration of 53.4 nmol/L in September [70]. These studies did not find a correlation between age and mean 25(OH)D in their database [70] which allows us to estimate the mean concentration of 25(OH)D in elderly in Switzerland by computing the weighted average of the two data sets, resulting in a mean concentration of 47.0 nmol/L. This estimation is in agreement with a previous estimation of 46 nmol/L in 1992 based on analyses of 3200 subjects [71] in Switzerland.

**Fig. 5:**
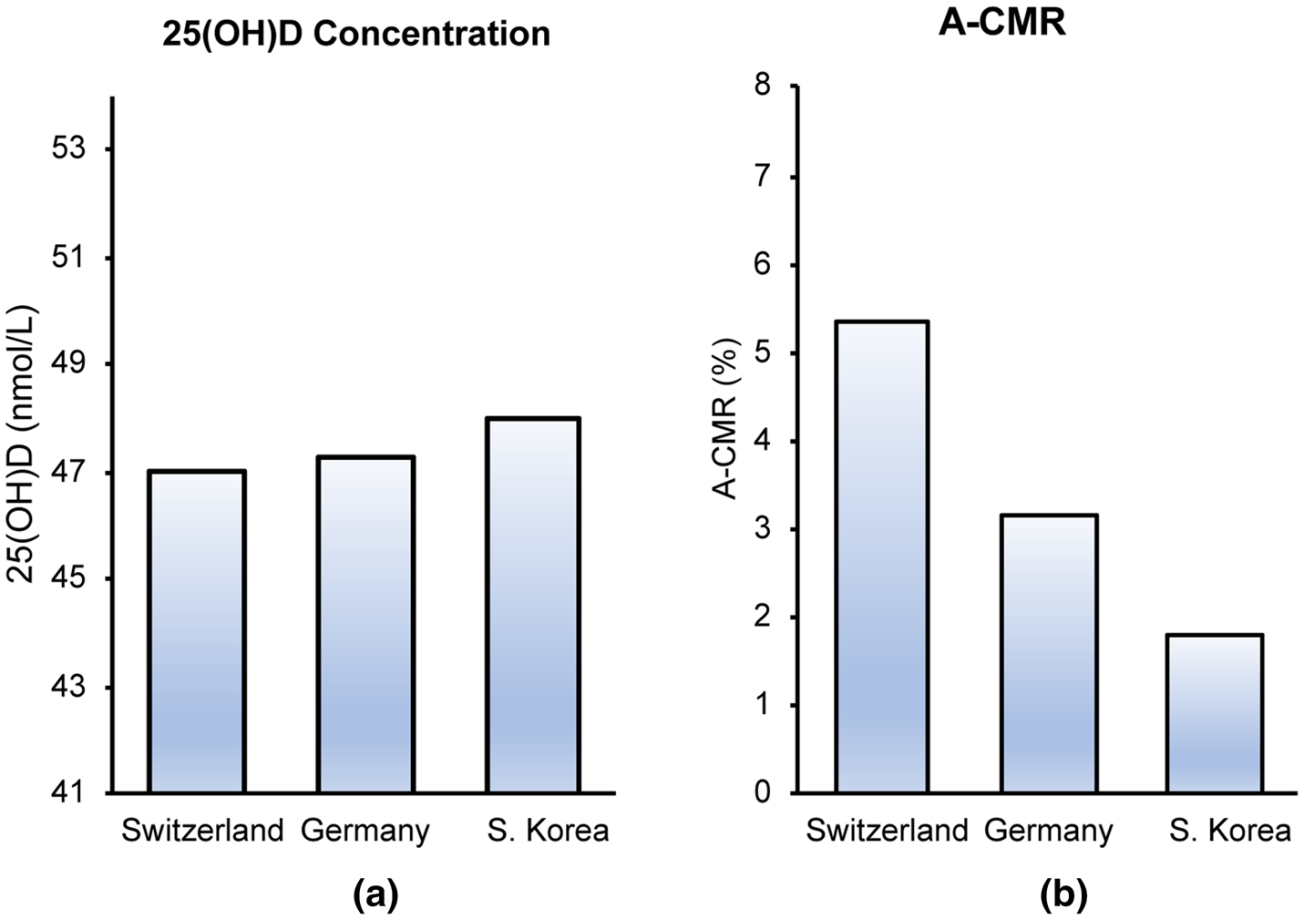
Mean 25(OH)D concentration in the elderly population in Germany [53, 68], Switzerland [70] and S. Korea [54]

The mean 25(OH)D concentration and A-CMR (shown in Fig. 5) indicate that S. Korea is reporting a lower A-CMR than Germany and Switzerland while also reporting a higher mean 25(OH)D among the elderly. However, the small number of countries and the narrow difference between the concentration of 25(OH)D in these three countries do not allow us to make a conclusion based on this result.

#### Possible impact of chronic factors on A-CMR

**Fig. 6:**
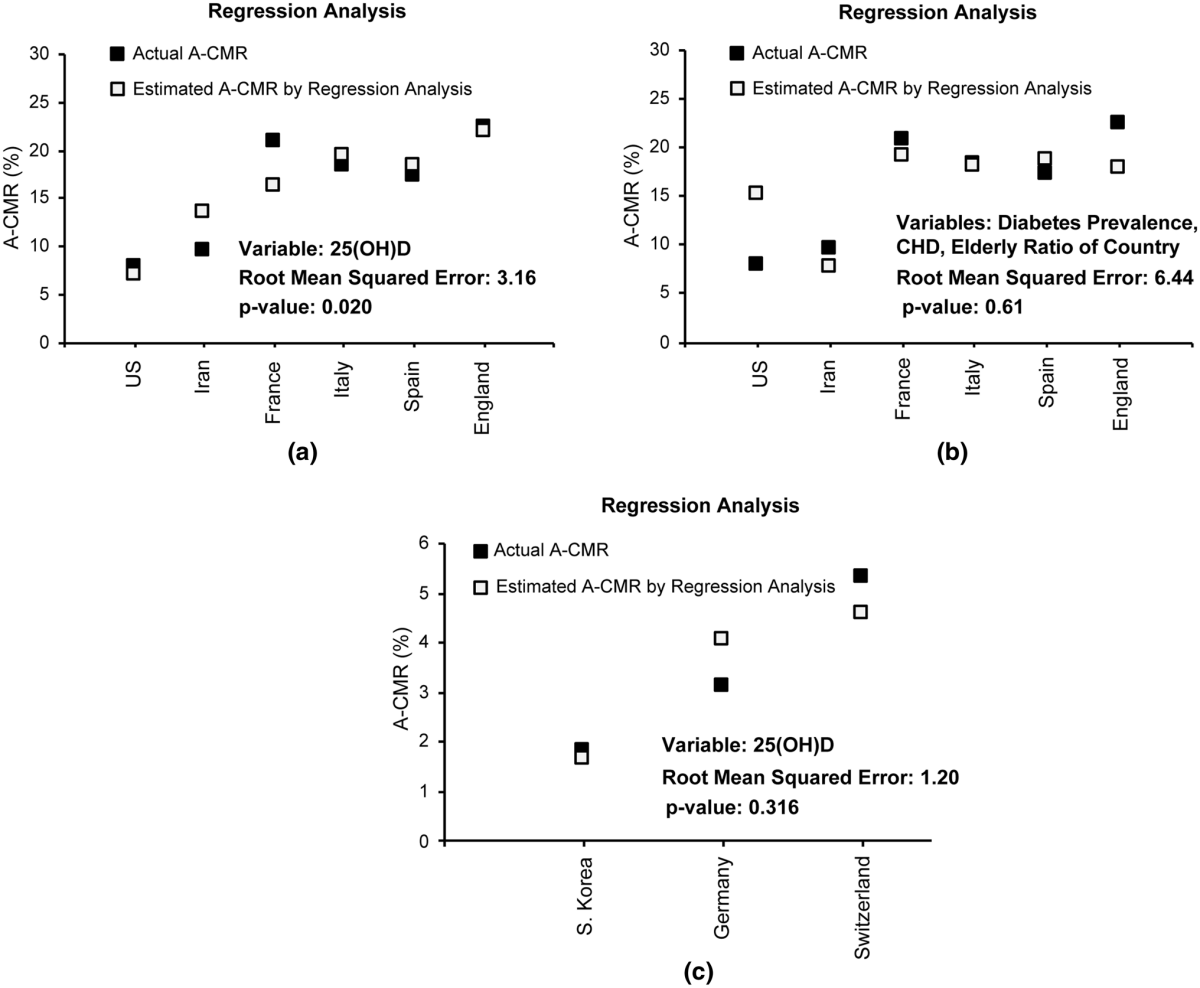
Regression analysis based on **a** 25(OH)D, **b** Diabetes prevalence among men and women (age-standardized), CHD death rate per 100,000 (age-standardized), and Elderly ratio (age ≥ 70 yo) in the countries with less aggressive screening strategy. **c** Regression analysis based on 25(OH)D in countries with more aggressive screening strategy

Mortality across a population may be impacted by health care availability and prevalence of COVID-19. Our multiple regression analysis determined a significant relationship (*p* value = 0.00023) between positivity and mortality across countries, but we did not see a statistically significant relationship between the number of beds per 10,000 and A-CMR. As positivity depends on the prevalence of COVID-19, we extended our analysis by incorporating an estimation of prevalence into the equation via introducing TAI. The impact of 25(OH)D and other chronic factors such as elderly ratio, CHD prevalence, high blood pressure prevalence, body to mass ratio, and diabetes prevalence on A-CMR was investigated via multiple regression analysis in the six countries with low TAI (Spain, Italy, Iran, France, the England, and the US). Our regression analysis on 25(OH)D and investigated chronic factors revealed that only 25(OH)D presented a statistically significant relation with A-CMR. A regression model based on only 25(OH)D (shown in Fig. 6a) across the countries with low TAI predicts A-CMR with a root mean squared error of 3.16 and presents a p value of 0.020 while the regression model created based on three chronic factors of diabetes prevalence (age-standardized), CHD death rate per 100,000 (age-standardized), and elderly ratio (chronic factors with smallest *p* value) can predict A-CMR with a root mean squared error of 6.44 and presents *p* value of 0.61 (Fig. 6b).

An inverse association is also investigated between 25(OH)D and A-CMR among the countries with high TAI and the regression modeling is shown in Fig. 6c. The small difference of the mean concentration of 25(OH)D between Germany, S. Korea, and Switzerland does not allow an evaluation of the link between 25(OH)D and A-CMR, however, it did enable us to further evaluate the impact of other chronic factors on A-CMR when the mean 25(OH)D of the elderly is similar between countries. We used regression analysis and did not find a statistically significant relationship between any of the other investigated chronic factors and A-CMR. Our analysis partially addresses the limitation that VitD associated with mortality due to its correlation with the underlying conditions such as diabetes, coronary heart disease, or age, however, we cannot exclude residual confounding factors.

### Disparity in confirmed, hospitalized and admitted to ICU cases across age groups

**Fig. 7:**
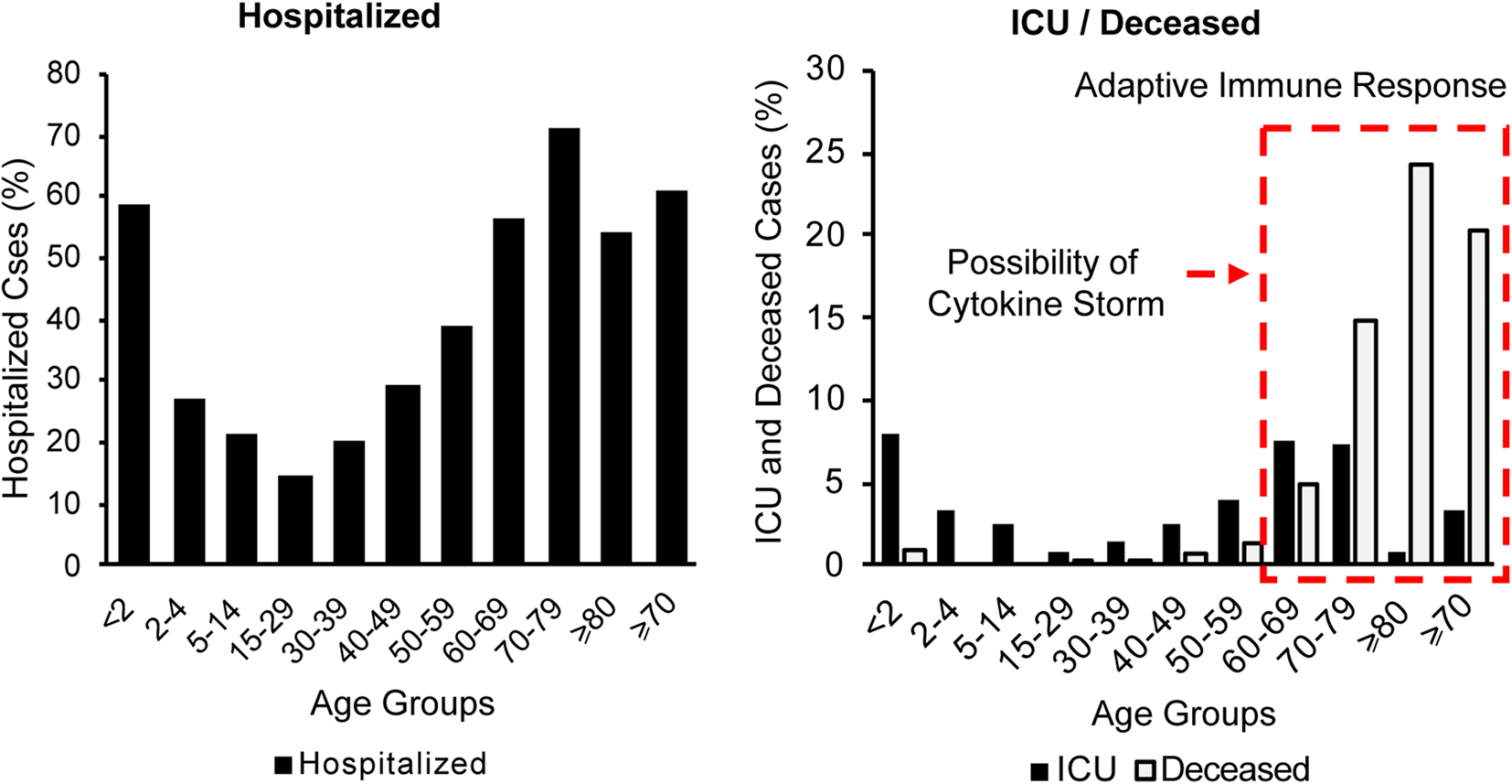
Age distribution of the **a** hospitalized, **b** admitted to ICU or deceased in Spain based on data from 145,429 cases [44]

The impact of aging on innate immunity may influence the body’s response against COVID-19. Age distribution of 145,429 patients in Spain, shown in Fig. 7, indicates an alarming impact of COVID-19 on the elderly [44]. In particular, 61% of the patients above 70 yo were hospitalized and 20% died. A possible explanation for this is that a weak innate immune system response to COVID-19 resulted in an elevated viral load, which then led to complications associated with hospitalization. Consequent overactivation of the adaptive immune system and high levels of cytokine production [9] could lead to complications that must be addressed in the ICU.

**Fig. 8:**
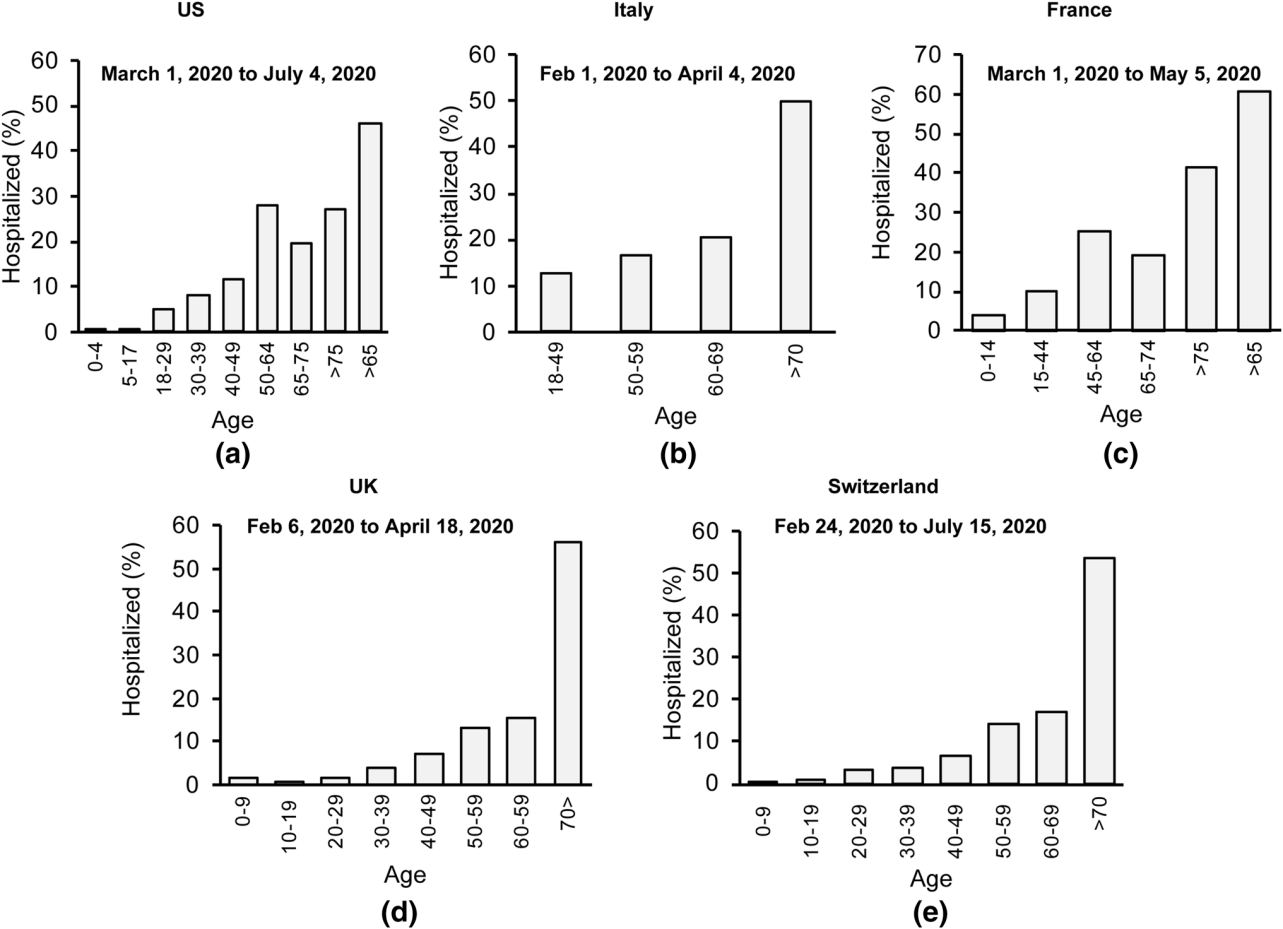
Age distribution of the hospitalized in **a** the USA [37, 38], **b** Italy [40], **c** France [39], **d** the UK [43] and **e** Switzerland [41, 42]

Our assessment of the age distribution of hospitalized patients in other countries suggests a similar pattern where elderly with age > 70 yo are disproportionately hospitalized due to COVID-19 as shown in Fig. 8.

**Fig. 9:**
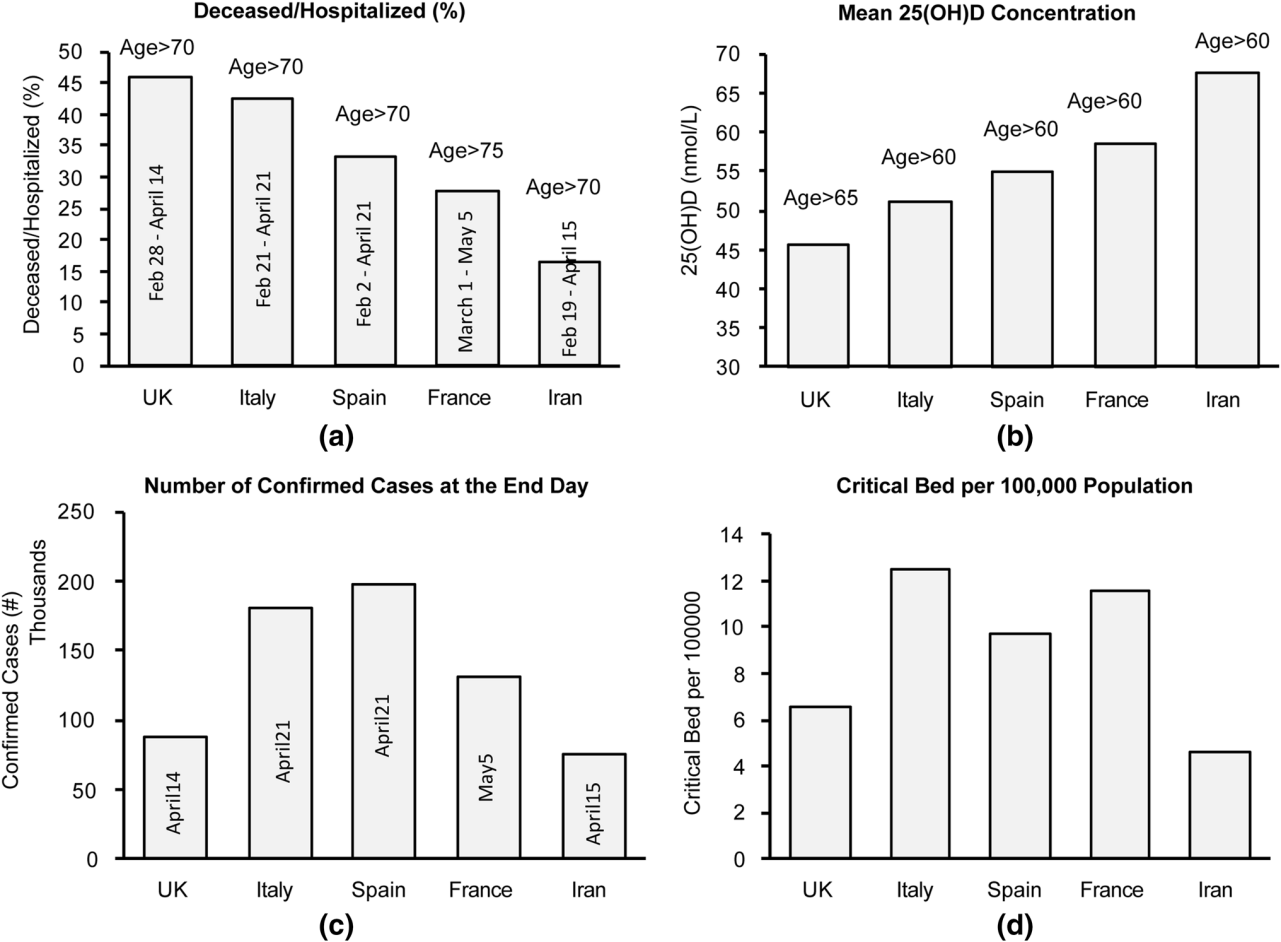
**a** Estimated death rate among hospitalized elderly patients, **b** estimated concentration of 25(OH)D in elderly, **c** total number of confirmed cases in the country at the end of study interval and **d** number of critical beds per 100,000 population in the UK [43], Italy [40], Spain [44], France [39], and Iran [45]

Cytokine storm and unregulated inflammation are expected to have a selectively greater impact on hospitalized patients and the elderly population among them. Many confounding factors in each population make an accurate assessment of the possible VitD impact on cytokine storm and unregulated inflammation reported in COVID-19 patients extremely difficult. To reduce the impact of some of these factors, we collected and estimated COVID-19 deceased ratio among hospitalized elderly populations in different countries (based on the available data) and the results are shown in Fig. 9a. 25(OH)D concentration of the elderly in each country is shown in Fig. 9b. For the elderly population, a lower ratio of deceased to the hospitalized cases is observed in the countries with higher 25(OH)D concentration. Regression analysis suggests an inverse correlation of *r* = − 0.92 with *p* value = 0.009.

There are other factors which still may have impacted the results of this analysis. We have intended to look at similar state of outbreak for each country. However, the number of confirmed patients in each country would not be identical and thus the number of hospital beds per capita may partially be accounted for. The cultural variation in hospitalizing the patients is another factor impacting these results. We are looking at the mortality of patients hospitalized in each country where the number of hospital beds becomes less important, however the number of intensive care units and ventilators available to the hospitalized populations in each country may impact these results. The number of confirmed cases in a country at the end of each study is representative of the hospital patient caseload in the period of analysis shown in Fig. 9c. The number of critical beds per 100,000 population in each country, shown in Fig. 9d, is a factor that may impact the mortality ratio in each country. Although these two variables are expected to partially impact the deceased/hospitalized ratio, our multi-regression analysis did not suggest any statistically significant relationship between them and the elderly mortality ratio shown in Fig. 9a.

To assess the impact of some confounding factors we investigated the number of ventilators in each country and also which countries reported a ventilator shortage in the period of this analysis. The UK with population of 67.9 million reported that they used 8000 of their 10,000 mechanical ventilators as of April 1, 2020 [72] while they also had access to an additional 1300 noninvasive ventilators [72]. Thus, we cannot suggest an extreme shortage of ventilators during the period of analysis is responsible for the higher mortality rate of the population in the UK compared to the other countries. France with a population of 65.2 million reported access to 5000 ventilators [73] while Switzerland with a notably smaller population of 8.6 million reported access to 750 ventilators [74]. Italy and Spain were the first two European countries that reported an unexpected outbreak and the media reported a shortage of ventilators in both countries during the time period in Fig. 9 [75]. A lower mortality rate could be reported in Italy and Spain with an access to sufficient number of ventilators in the period of this analysis. The data for Iran are based on an epidemiological study from a single center in Tehran which analyzed data for 2968 hospitalized COVID 19 patients (out of 12,870 patients) from February 19, 2020, to April 15, 2020 [45]. The deceased to hospitalized ratio can be different in other provinces or centers due to possible variations in the resources and caseloads of patients. Figures from the CDC Morbidity and Mortality Weekly Report of March 18, 2020, suggests 24 out of 109 elderly (age > 75 yo) were deceased from February 12, 2020, to March 16, 2020, in the US which amounts to 22% of the elderly population [76]. We did not include this result in Fig. 9, as it was prepared based on limited data from 508 hospitalized patients in entire country with a biased distribution of reported cases of hospitalization toward the end of study [76]. The design of our analysis partially addresses and reduces, but cannot exclude, these residual confounding factors.

### CRP and severe COVID-19

Table 1 shows the risks of severe and mild COVID-19 under different CRP levels, based on clinical data from 793 confirmed COVID-19 patients in China (up to 52 hospitals in 30 provinces) [31]. According to this dataset, patients with severe COVID-19 have a higher incidence of high CRP (81.5%, 110 cases out of 135) than those with a mild form of the disease (56.5%, 371 cases out of 658) suggesting an odds ratio (OR) of 3.4 with 95% confidence interval (95% CI) (2.15 to 5.4). This is evidence for a higher likelihood of unregulated inflammation associated with cytokine storm among the patient with severe COVID-19.

**Table 1:** The risks of severe and mild COVID-19 under different CRP levels, based on data reported by [31]

	Number of events/total patients (risk)
Risk of high CRP	481/793 (61%)
Risk of low CRP	312/793 (39%)
Risk of high CRP given severe COVID-19	110/135 (81%)
Risk of low CRP given sever COVID-19	25/135 (19%)
Risk of severe COVID-19 given high CRP	110/481 (23%)
Risk of severe COVID-19 given low CRP	25/312 (8%)
Risk of high CRP given mild COVID-19	371/658 (56%)
Risk of low CRP given mild COVID-19	287/658 (44%)

### Possible association of VitD deficiency with CRP and cytokines

Production of IL-6 by monocyte, dendritic cells, and macrophage in patients with severe COVID-19 leads to systematic pro-inflammatory cytokines and CRP production [77] and in the absence of anti-inflammatory cytokines may lead to a high-grade inflammation and cytokine storm. Although CRP is a nonspecific marker, it becomes more specific to bioactivity of IL-6 and formation of a cytokine storm [26, 27, 78] in patients with severe COVID-19 [77].

Clinical data reported by Guan et al. (summarized in Table 1) indicate that the risk of high CRP in severe COVID-19 patients is 44.5% higher than patients with mild COVID-19 [31]. VitD deficiency leads to the production of cytokines such as tumor necrosis factor (TNF)-α and IL-1β through the intercellular activity of calcium [79] which may cause inflammations and elevates CRP. This may explain the simultaneous attenuation of CRP and inflammatory cytokines (CD4( +) IFN-γ) in hemodialysis patients after calcitriol treatment [80], or elevation of both CRP and cytokines in severe COVID-19 patients [31]. A recent study has shown that VitD can alter the bioactivity of IL-6 to induce more anti-inflammatory cytokines, such as IL-10, instead of pro-inflammatory cytokines such as IL-17, which is expected to lead to the reduction of CRP [81].

**Fig. 10:**
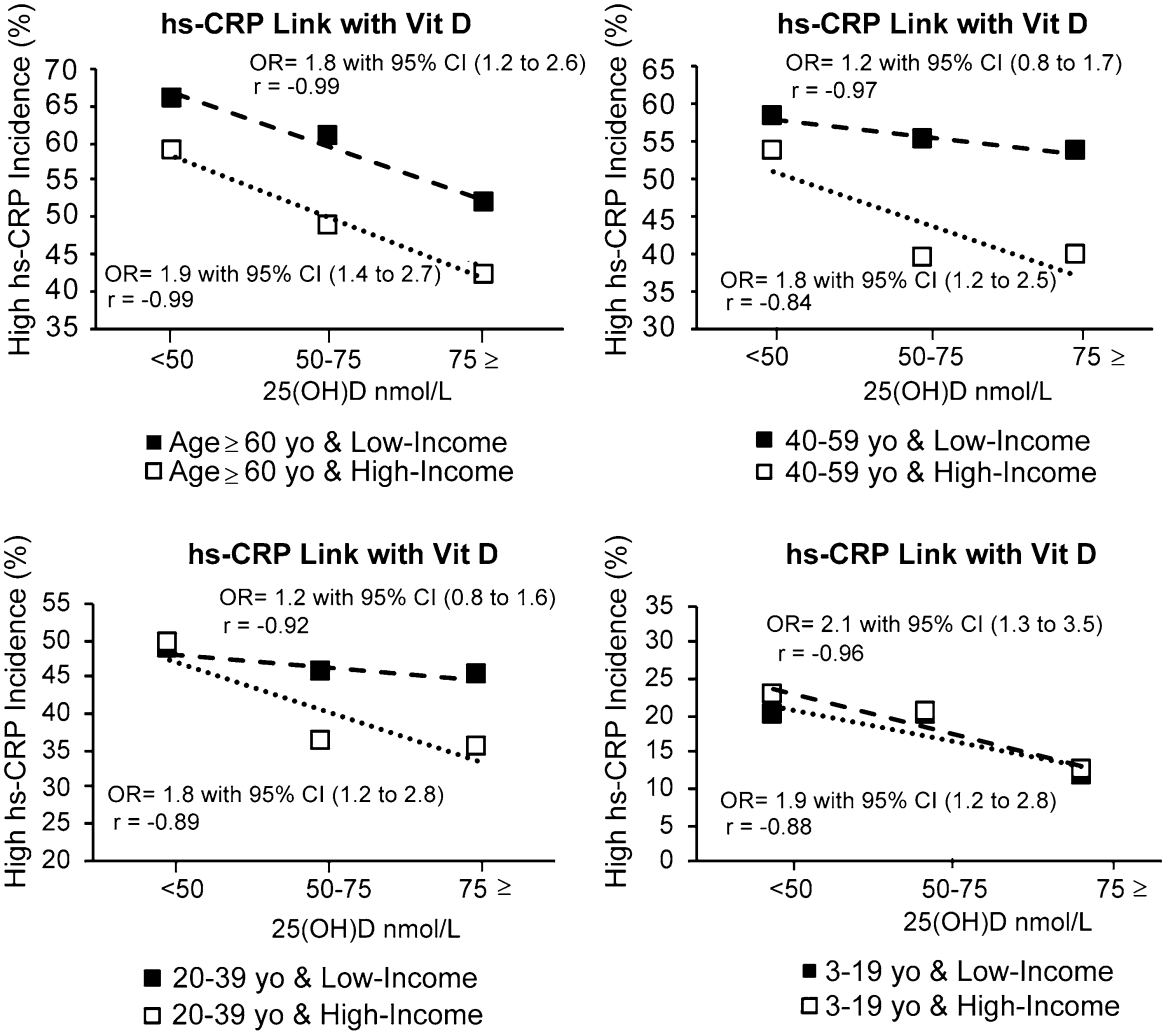
High hs-CRP and possible low-grade inflammation association with VitD status

Our analysis of VitD status and high hs-CRP in 8345 participants with similar age groups and family income status from NHANES, 2009–2010, shown in Fig. 10, suggests subjects with VitD deficiency have 34% (age ≥ 60 yo), 22% (20 yo ≤ age < 40 yo), and 21% (40 yo ≤ age < 60 yo) more incidence of high hs-CRP, respectively, than patients with normal VitD status.

**Fig. 11:**
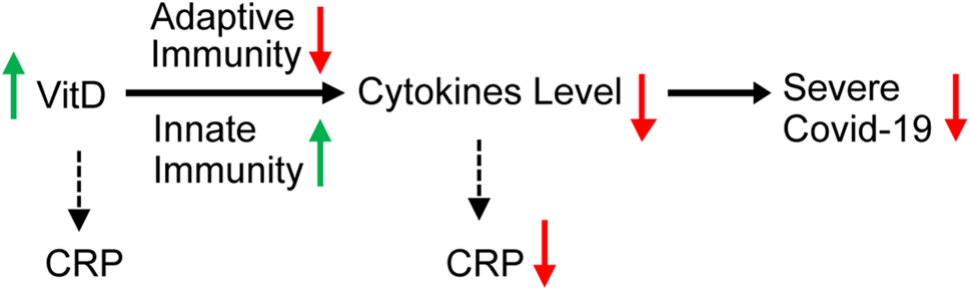
Possible impact of improving VitD status on the reduction of cytokines and CRP

This result suggests the production of more low-grade inflammation in patients with VitD deficiency than patients with normal VitD status and includes inflammations caused by bioactivation of IL-6 and production of pro-inflammatory cytokines. CRP is widely considered as a surrogate of IL-6 bioactivity [26, 27, 78] and the role of IL-6 in inducing pro-inflammatory cytokines and the development of cytokine storm in COVID-19 patients indicates the importance of CRP in the assessment of the related complications. CRP as a surrogate of IL-6 bioactivity may be a more accurate indicator of pro-inflammatory cytokines than IL-6 concentration, as IL-6 bioactivity may change in the presence of VitD [81]. Thus, we suggest a possible role for VitD in reducing pro-inflammatory cytokine levels and CRPs based on retrospective data and indirect evidence (shown in Fig. 11).

Although we have not analyzed the CRP data in populations from other countries, there is increasing evidence showing the inverse association between VitD concentration and inflammatory markers such as hs-CRP. A cross-sectional study of 253 elderly (51 yo to 77 yo) from North Western Adelaide Health Study in Australia reported an inverse correlation (*β* =  − 0.14, *p* value = 0.03) between the low concentration of 25(OH)D3 and hs-CRP [82]. Another study of 2723 subjects (25 yo to 88 yo) in northeast Germany reported a U-shape association between VitD and hs-CRP where an inverse correlation was observed in low VitD regions (25(OH)D < 70 nmol/L). This study also demonstrated an inverse correlation between VitD and other inflammatory markers such as fibrinogen (*p* value < 0.01) [83]. Analysis of VitD and hs-CRP concentration in 10,118 subjects during the sixth Tromsø Study in Norway indicated a negative association between VitD and hs-CRP [84]. Observational analysis of 3586 subjects in Finland based on Northern Finland Birth Cohort 1966 (NFBC1966) reported an inverse association between 25(OH)D and hs-CRP [85]. A cross-sectional study of 147 obese subjects in Italy (89 female) showed a significant inverse correlation between 25(OH)D and hs-CRP(*r* = − 0.31; *p* = 0.043), IL-6 (*r* = − 0.49; *p* = 0.003) and TNF-α (*r* = − 0.61; *p* = 0.001) suggesting the link between 25(OH)D and low-grade systematic inflammation in obese subjects [86]. Analysis of medical data from 9649 subjects age ≥ 55 yo in Rotterdam in the Netherlands indicated an inverse association between 25(OH)D and hs-CRP. Assessment of VitD association with hs-CRP in patients with renal cell carcinoma (RCC) in China shows an inverse correlation between VitD and hs-CRP (*r* =  − 0.25, *P* < 0.05) [87]. Majority of the patients with 25(OH)D < 50 nmol/dL show high hs-CRP with average hs-CRP of 2 mg/dL while the majority of patients with 25(OH)D > 50 nmol/dL are presenting a hs-CRP < 1 mg/dL [87].

Complete control of all unknown parameters in clinical studies investigating the impact of VitD supplementation on hs-CRP is extremely difficult. Most studies have reported that VitD supplementation is inversly associated with hs-CRP levels while others did not see the impact of the supplementation. The result of a meta-analysis targeting 10 trials from 924 human subjects in countries such as the US, Norway, Finland, and Iran suggested VitD supplementation may reduce hs-CRP by 0.108 mg/dL with 95% CI of (− 0.213 to − 0.003) where the decrease may be more notable in subgroups with higher hs-CRP levels (> 0.5 mg/dL) and drops up to 0.221 mg/dL with 95% CI of (− 0.350 to − 0.092) [88]. Another meta-analysis focusing on subjects with type-2 diabetes also concluded an inverse correlation between VitD and hs-CRP (hs-CRP drops by 0.034 mg/dL). The optimum dose of VitD supplementation that leads to the highest impact on hs-CRP has not been identified and further research is required in this area.

## Discussion

Our analysis of large-scale data suggests a possible link between VitD deficiency and A-CMR among countries with similar testing strategies. This is evidence supporting the role of VitD in enhancing the immune system and potentially reducing the complications associated with cytokine storm and unregulated inflammation in elderly patients with severe COVID-19. There is emerging evidence supporting a possible protective impact of VitD on severe COVID-19. A recent study comparing mortality in countries in the Southern and Northern Hemispheres, also supports the possible association of VitD with COVID-19 [89]. A study of 25(OH)D data from a cohort of patients from Switzerland shows 25(OH)D data in 27 patients with PCR-positive for SARS-CoV-2 (median 25(OH)D of 27.75 nmol/L) is significantly (*p* value = 0.004) lower than the 80 patients with negative PCR-positive for SARS-CoV-2 (median 25(OH)D of 61.5 nmol/L) [90]. This study also showed that 25(OH)D in the 18 PCR-positive patients with age > 70 yo (median 25(OH)D of 23.25 nmol/L) is also significantly (*p* value = 0.037) lower than the 43 PCR-negative patients (median 25(OH)D of 57.75 nmol/L) [90]. Additionally, analysis of a recent laboratory data of 4314 subjects tested for COVID-19 at the University of Chicago Medicine shows a disproportionally higher COVID-19 positivity rate for patients with VitD deficiency. 32 out of 140 tested subjects with VitD deficiency (25(OH)D < 50 nmol/dL) were infected with SARS-CoV-2 while 39 out of 278 subjects without VitD deficiency were infected with the virus. These results present indirect evidence that hospitalization ratio due to COVID-19 may be notably higher for patients with VitD deficiency. Recent findings from a study investigating the risk of COVID-19 for Parkinson’s Disease (PD) patients Living in Lombardy, Italy suggests the possibility of the protective impact of VitD supplementation against infection with SARS-CoV-2. Interviews of subjects show 12.4% of COVID-19 patients (13 out of 105 PD subjects) were taking VitD supplementation while 22.5% (316 out of 1381 PD subjects) of the unaffected subjects were taking VitD supplementation [91].

Our analysis mainly focused on the possible impact of VitD on the reduction of cytokine storm which can reduce mortality among the elderly population. Severe COVID-19 patients show a notable elevation of inflammatory cytokines such as IL-2R, IL-6, granulocyte colony-stimulating factor (GCSF), macrophage chemotactic protein-1 (MCP1), macrophage inflammatory protein (MIP)1A, TNF-α and anti-inflammatory compounds such as CRP [10, 92]. Complications associated with cytokine storm include Acute Respiratory Distress Syndrome (ARDS), exacerbation of the effects of pneumonia, acute kidney failure, acute heart failure, and rhabdomyolysis [31] which may become fatal. Elderly patients with an aberrant innate immune system may be subject to elevated viral load [8, 93] followed by misfiring and over-activation of their adaptive immune system through differentiating CD8 + T cells into Cytotoxic T Lymphocytes (CTLs) [94] potentially resulting in a cytokine storm. Of particular note is that the time interval for the development of a substantial adaptive immune response, approximately 7 days after development of symptomatic disease, is consistent with the time course of COVID-19 mortality [10, 31]. The reported potential role of ibuprofen in worsening COVID-19 treatment [95] might also be partially explained by its suppression of innate immunity [96, 97] which may lead to a higher viral load and consequent overactivation of the adaptive immune system which again may become fatal in elderly patients [98, 99]. Even moderate lung damage due to a weak cytokine storm could lead to hypoxemia that in turn results in mortality due to underlying conditions. Further, the possible role of dexamethasone in reducing the cytokine storm is recently shown to reduce the mortality rate of COVID-19 [100].

Multiple studies have demonstrated the role of VitD in regulating the immune system. VitD may suppress cytokine production by simultaneously boosting the innate immune system and reducing the overactivation of the adaptive immune system in response to increased viral load [11, 13]. A recent study showed that the CD4 + T-helper cells in the bronchoalveolar lavage fluid (BALF) of COVID-19 patients induce significant changes in gene expression and that the upregulated genes were enriched in pathways associated with pro-inflammatory cytokine of IFN-γ [81]. These researchers further discovered that the anti-inflammatory IL-10 is notably (fourfold) lower in samples from COVID-19 patients compared to the healthy control. They showed that VitD regulates CD4 + T-helper response to suppress gene expression of pro-inflammatory cytokines such as IFN-γ and IL-17 and induce anti-inflammatory cytokines such as IL-10. The group further reported that VitD suppresses type 1 cytokines such as Interferon Gamma (IFNG) and type 3 cytokines such as IL17A, IL17F, IL22, and IL26 [81]. Although this study reported that VitD induced gene expression of IL-6, they showed the biochemistry of IL-6 changes in the presence of VitD to induce anti-inflammatory cytokine IL-10 instead of pro-inflammatory cytokine, IL-17 [81].

VitD deficiency is more prevalent among the elderly and African-Americans but appears in every population group. While this epidemiological study provides compelling correlational evidence, we acknowledge that it does not speak to causation. Indeed, while low VitD levels have been associated with a variety of conditions (coronary artery disease, diabetes, cancers, autoimmune, obesity and others) [24], many randomized controlled trials on VitD supplementation have been disappointing [101, 102]. This may be related to trial issues including the time frame of intervention, VitD receptor polymorphisms, or the need to consider complementary or synergistic interventions, but underscores the need for caution. An alternative explanation for our findings may be that low VitD status is a marker for underlying health issues which are known to be a risk factor in COVID-19 fatalities. Balancing this out is the strong biological/mechanistic plausibility for VitD's direct role in COVID-19 mitigation. This highlights the urgency for future randomized controlled trials.

Further, one important limitation of the present country-level analysis is the assumption that VitD levels in COVID-19 patients follow the same distribution with subjects in other previous VitD studies. We did not have access to VitD status and cytokine levels in individual COVID-19 patients before and after infection. In other words, we do not have the data to suggest that VitD is therapeutic. Leveraging available data, we illustrated evidence for possible association between VitD and unregulated cytokines and CRP (a surrogate of cytokine storm). In addition, the difference in age range, ethnicity, gender, social status, geographic latitude, measurement variations, the season of sample collection, and year of study may impact the reported value of VitD status in different studies. We reduced some of these impacts by analyzing data from more recent studies which have collected a large number of samples through the entire year, however the distribution of samples collected in each season may not be equal across all studies. Different laboratory methods used in the estimation of 25(OH)D concentration in different studies across the world is another limiting factor. Our data could not exclude residual confounding factors. The intrinsic cross-sectional nature of this study does not prove a relationship between VitD, CRP levels, cytokine storm, and severe COVID-19. VitD data have been collected from different sources and variation between and within different studies introduces variations in the data. Another important limitation of this study is that crude mortality data is used instead of age-specific mortality data. The onset of COVID-19 for confirmed cases is unknown and is assumed to be similar for all subjects. In addition, other underlying conditions associated with the populations at risk of VitD deficiency makes it more challenging to assess the actual impact of VitD in comparison to other factors. These limitations can be addressed by following VitD and COVID-19 status in individual patients within a given population. Such data, however, are currently unavailable. The link between VitD and the probability of severe COVID-19 and associated mortality that is indicated by this work may serve as an impetus for such studies.

## Conclusion

Large-scale data show that screening strategies notably impact A-CMR, as countries with aggressive COVID-19 screening show decreased A-CMR. Our analysis of mean 25(OH)D of elderly in countries with similar testing strategies suggests a possible role of VitD in reducing A-CMR which provides evidence supporting the impact of VitD on the immune system and reducing unregulated cytokine production and inflammation. Our hypothesis on the role of cytokine storm and unregulated inflammation in COVID-19 complications is consistent with findings such as an increase in the rate of complications with age, low rate of complications in children, and adverse outcomes with ibuprofen, and it might be of interest to study VitD’s impact on COVID-19 in controlled observational or clinical trials.

CRP is the marker for bioactivity of IL-6 which plays a major role in the development of cytokine storm [26, 27, 77]. Our analysis of hs-CRP in healthy subjects indicated an OR of 1.8 with 95% CI (1.2 to 2.6) among the elderly (age ≥ 60 yo) in low-income families and an OR of 1.9 with 95% CI (1.4 to 2.7) among the elderly (age ≥ 60 yo) in high-income families. This is indirect evidence supporting the association of VitD with cytokines and unregulated inflammation, as these are partially responsible for the elevation of CRP. In severe COVID-19 cases, cytokine storm notably increases the production of CRP, and as such a stronger correlation between cytokine storm and high CRP is achieved. Patient-level data shows a notable OR of 3.4 with 95% CI (2.15 to 5.4) for high CRP in severe COVID-19 patients. Based on retrospective data and indirect evidence, we see a possible role of VitD in reducing complications attributed to cytokine storm and unregulated inflammation however we emphasize that we do not have the patient-level data to suggest that VitD is therapeutic. Our conclusion is that future studies of the role of VitD in reducing cytokine storm and COVID-19 mortality are warranted.
