# Correction: Evidence for possible association of vitamin D status with cytokine storm and unregulated inflammation in COVID-19 patients

**DOI:** 10.1007/s40520-023-02627-0

**Published:** 2023-12-08

**Authors:** Ali Daneshkhah, Vasundhara Agrawal, Adam Eshein, Hariharan Subramanian, Hemant Kumar Roy, Vadim Backman

**Affiliations:** 1 Department of Biomedical Engineering, Northwestern University, Evanston, IL USA; 2 Boston Medical Center, Boston, MA USA

**Correction: Aging Clinical and Experimental Research (2020) 32:2141–2158** 10.1007/s40520-020-01677-y

In the Acknowledgements section of this article the grant number relating to National Institutes of Health given was incorrectly given as

The authors would like to thank Benjamin D Keane for his assistance in preparing the manuscript. The authors would also like to acknowledge generous support from the Carinato Charitable Foundation, Mark and Ingeborg Holliday, Kristin Hudson and Rob Goldman, and Ms. Susan Brice and Mr. Jordi Esteve.

and should have been

The authors would like to thank Benjamin D Keane for his assistance in preparing the manuscript. The authors would also like to acknowledge generous support from the Carinato Charitable Foundation, Mark and Ingeborg Holliday, Kristin Hudson and Rob Goldman, and Ms. Susan Brice and Mr. Jordi Esteve. In addition, the authors would also like to acknowledge funding from the National Institutes of Health (NIH), through grant #R01CA225002.

The original article has been corrected.

